# Small‐spot intensity‐modulated proton therapy and volumetric‐modulated arc therapies for patients with locally advanced non‐small‐cell lung cancer: A dosimetric comparative study

**DOI:** 10.1002/acm2.12459

**Published:** 2018-10-17

**Authors:** Chenbin Liu, Terence T. Sio, Wei Deng, Jie Shan, Thomas B. Daniels, William G. Rule, Pedro R. Lara, Shawn M. Korte, Jiajian Shen, Xiaoning Ding, Steven E. Schild, Martin Bues, Wei Liu

**Affiliations:** ^1^ Department of Radiation Oncology Mayo Clinic Phoenix AZ USA; ^2^ Department of Biomedical Informatics Arizona State University Tempe AZ USA

**Keywords:** intensity‐modulated proton therapy, interplay effects, lung cancer, volumetric‐modulated arc therapy

## Abstract

**Purpose:**

To compare dosimetric performance of volumetric‐modulated arc therapy (VMAT) and small‐spot intensity‐modulated proton therapy for stage III non‐small‐cell lung cancer (NSCLC).

**Methods and Materials:**

A total of 24 NSCLC patients were retrospectively reviewed; 12 patients received intensity‐modulated proton therapy (IMPT) and the remaining 12 received VMAT. Both plans were generated by delivering prescription doses to clinical target volumes (CTV) on averaged 4D‐CTs. The dose‐volume‐histograms (DVH) band method was used to quantify plan robustness. Software was developed to evaluate interplay effects with randomized starting phases of each field per fraction. DVH indices were compared using Wilcoxon rank sum test.

**Results:**

Compared with VMAT, IMPT delivered significantly lower cord D_max_, heart D_mean_, and lung V_5 Gy[_ _RBE__]_ with comparable CTV dose homogeneity, and protection of other OARs. In terms of plan robustness, the IMPT plans were statistically better than VMAT plans in heart D_mean_, but were statistically worse in CTV dose coverage, cord D_max_, lung D_mean_, and V_5 Gy[_ _RBE__]_. Other DVH indices were comparable. The IMPT plans still met the standard clinical requirements with interplay effects considered.

**Conclusions:**

Small‐spot IMPT improves cord, heart, and lung sparing compared to VMAT and achieves clinically acceptable plan robustness at least for the patients included in this study with motion amplitude less than 11 mm. Our study supports the usage of IMPT to treat some lung cancer patients.

## 1 INTRODUCTION

Lung cancer is the leading cause of cancer death among both men and women in the United States. Non‐small‐cell lung cancers (NSCLC) account for about 85% of lung cancer cases.^1^, ^2^ Radiotherapy combined with chemotherapy is standard treatment for stage III NSCLC patients with unresectable tumors, but the potential toxic effects of radiation limit the feasibility for delivering adequate tumoricidal dose to targets in most patients.^3^, ^4^ With photon radiation and concurrent chemotherapy, the long‐term results from RTOG 0617 reported 5‐year overall survival (5‐year OS) of 32.1% (standard dose arm with 60 Gy) and 23% (high dose arm of 74 Gy) for unresectable NSCLC patients.^5^ The fact that dose escalation has led to worse overall survival is possibly due to higher cardiac toxicity.^4^, ^6^ The improvement of overall survival would require the minimization of incidental radiation dose to critical normal structures.

Volumetric‐modulated arc therapy (VMAT) is an advanced form of intensity‐modulated radiation therapy (IMRT) that can deliver a precisely sculpted dose distribution using a single or multi‐arcs.^7^ It has gained popularity in treating lung cancer patients due to its superior dose coverage, decreased radiation‐induced pneumonitis, and shorter delivery time compared to conventional static‐field IMRT.^8^, ^9^, ^10^, ^11^ On the other hand, due to the sharp falloff of dose deposition distal to the Bragg peak, proton therapy has great potential to provide highly conformal tumor target coverage while sparing adjacent organs at risk (OARs), such as heart, lungs, spinal cord, and esophagus.^12^, ^13^ Proton therapy is used in three different modalities: passive‐scattering proton therapy (PSPT), uniform scanning proton therapy (USPT), and intensity‐modulated proton therapy (IMPT). Recently, Chang et al.^14^ published a phase 2 study of high dose PSPT (74 Gy[RBE]) and concurrent chemotherapy for unresectable stage III NSCLC. They reported 5‐year OS of 29% with very low rates of toxicities. It seemed that high dose PSPT tended to have better 5‐year OS than the high dose photon therapy, but still slightly worse outcomes than the standard dose photon therapy if we compared this clinical trial data to RTOG 0617. Therefore, they suggested the use of IMPT to further improve the dose conformality and reduce doses to nearby OARs.^15^, ^16^

Unfortunately, IMPT is subject to increased uncertainties for moving targets compared with PSPT and USPT.^17^, ^18^, ^19^ Previous studies used proton pencil beam machines with in‐air sigma at the isocenter as large as 6~15 mm (depending on proton energy) to treat NSCLC cancer.^15^, ^16^ In this study, we defined these machines as large‐spot proton machines compared to the proton pencil beam machines with in‐air sigma at the isocenter of 2~6 mm (depending on proton energy), which we defined as small‐spot proton machines for the purpose of this study. There is a concern that IMPT with small‐spot size may not be a good option for lung cancer treatments with large motions, due to the concerns of uncertainties and interplay effects.^20^ A study by Chang et al. suggested that thoracic malignancies with tumor motion larger than 5 mm may not be safely treated using IMPT.^21^ Other studies suggested that IMPT treatment may be used for tumors with motion larger than 5 mm, but it would be negatively impacted by interplay effects, especially for small‐spot IMPT.^22^, ^23^, ^24^, ^25^, ^26^, ^27^, ^28^, ^29^, ^30^, ^31^, ^32^, ^33^ There are some studies that reported the limited impact of uncertainties and interplay effects in robustly optimized IMPT for stage III NSCLC.^34^, ^35^ There are no reports about dosimetric comparison between small‐spot IMPT and VMAT for NSCLC patients in term of plan quality, plan robustness in the face of uncertainties, and interplay effects.

IMPT with small‐spot sizes has been used to treat non‐moving targets for years. However, for moving targets such as lung cancer, previous researchers did demonstrate that small‐spot IMPT could improve the treatment plan quality.^36^ However, a simulation study showed that small‐spot IMPT (*σ*: 2~4 mm) could be less robust toward motion and interplay effects than large‐spot IMPT (*σ*: 8~17 mm).^28^ Larger number of spots will be needed to cover the same target volume if small‐spot proton machine was applied, which was also reported in a recent study.^37^ In the same study it was stated that interplay effects should be considered before IMPT treatment plan was delivered to lung cancer patients.^37^

Majority of the new proton centers being developed are equipped with spot scanning beam with small‐spot size (in‐air sigma at the isocenter as large as 2~6 mm) only. Currently, there are no studies sharing clinical experience in radiation oncology community concerning the treatment of stage III NSCLC patients with small‐spot IMPT. In this study, we reported the procedure implemented at our institution for small‐spot IMPT in the treatment of NSCLC patients. The study focused on the evaluation of plan quality, robustness and interplay effects, and compared the dosimetric parameters of small‐spot IMPT and VMAT.

## 2 MATERIALS AND METHODS

### 2.A Patient selection

We retrospectively reviewed 12 unresectable stage III NSCLC patients treated with IMPT consecutively between March 2016 and June 2017 at our institution. In addition, we retrospectively reviewed 12 selected stage III NSCLC patients treated by VMAT in the same time period at our institution. All plans used in this work were the clinically applied.

The patients included in this study were carefully selected by experienced physicists from the existing database of treated patients to ensure that the patients from the two treatment groups did not show significant differences in age, motion amplitude, or prescription doses (Table 1). However, the tumor size of patients treated by IMPT was significantly larger than that of patients treated with VMAT. All patients were staged using PET/CT and brain CT scans to rule out metastatic disease. All patients had an Eastern Cooperative Oncology Group (ECOG) performance status ≤2 and were definitively treated with radiation therapy with curative intent. None of the patients had implanted cardiac devices.

**Table 1:** Patient characteristics between the two treatment groups

	IMPT	VMAT	*P*‐value
Patient number	12	12	
Age at treatment (yr)			0.28
Median (Range)	74 (59–83)	70 (49–84)	
Gender			
Male, No.	5 (41.7%)	7 (58.3%)	
Tumor volume (cm^3^)			0.02
Median (Range)	257.1 (47.4–470.0)	98.0 (43.3–584.0)	
Motion amplitude (mm)			0.79
Median (Range)	6.5 (3.0–11.0)	6.3 (1.0–11.0)	
Prescription dose (Gy[RBE])			0.86
Median (Range)	60 (34–66)	60 (45–60)	

### 2.B Patient simulation and immobilization

All patients were simulated using four‐dimensional computed tomography (4D CT) in the supine position. Before image acquisition, the patient thorax was immobilized using Orfit board (Orfit Industries, Wijnegem, Belgium) and thermoplastic masks. The respiratory motion amplitude was defined by measuring the largest tumor mass center displacement in the three canonical directions in all 10 phases of the 4D CT. All patients selected for this study had motion amplitudes smaller than 11 mm. The 4D CT data sets were transferred to a commercial treatment planning system (Eclipse™, Varian medical system, Palo Alto, CA, USA) for localization of targets and contouring of OARs.

### 2.C Target and normal tissue definition

Treatment targets were defined as follows. Co‐registration with contrast enhanced CT scans and/or PET scans were used in identifying the gross target volume (GTV). The internal gross target volume (IGTV) was designed to encompass the extent of GTV motion in all phases of 4D CT. The clinical target volume (CTV) was formed by isotropic expansion of the IGTV by 5–10 mm (typically 7–8 mm). The value of margin expansions were based on the pathology of tumors and determined by experienced radiation oncologists. The CTV was adjusted based on patterns of potential tumor extent and anatomic boundaries such as vertebral body, chest wall, and heart, etc. Planning target volumes (PTVs) formed by 5 mm uniform expansion of CTVs were used for plan optimization and evaluation in VMAT. All normal tissues were contoured on the 4D averaged CT. CT artifacts were overridden using HU values sampled nearby.

### 2.D Treatment planning

IMPT treatment planning generally followed the treatment planning guidelines recommended by the Particle Therapy Co‐Operative Group (PTCOG) Thoracic and Lymphoma Subcommittee.^20^ The proton beam scanning machine for IMPT treatment was commissioned to have an energy‐dependent spot size (in‐air *σ*) of 2 mm to 6 mm and a fixed spot spacing of 5 mm was chosen in treatment planning. Discrete proton energies (from 71.3 to 228.8 MeV) were selected to minimize the ripple in the spread out Bragg peak (SOBP) dose distributions along the beam direction. VMAT treatment was administered using CLINAC machines (Varian Medical System, Palo Alto, CA, USA).

All IMPT plans were generated on the averaged 4D CT with IGTV density override (HU = 50). During the initial spot arrangement, an additional 7 mm margin expansion based on the PTV was used in the IMPT planning to ensure that there was at least one spot outside of the PTV to generate a possible homogeneous dose distribution within the PTV.

In most cases, two or three beams were used in IMPT. Beam directions in IMPT were chosen by dosimetrists with the help of experienced physicists if needed to minimize the impact of motion and spare normal tissues. Ten of twelve IMPT plans required single field optimization (SFO). If the SFO plan could not meet dosimetric and robustness requirements, a multiple field optimization (MFO) plan using robust optimization was generated. The final plan was chosen by an experienced radiation oncologist after careful evaluation of plan quality, plan robustness, and interplay effects.

For IMPT plans, two verification plans were generated by recalculating the dose on the exhale and inhale 4D CT phases (without the density override) to evaluate the impact of respiratory motion. The original plan was adjusted until the verification and original plan dose distributions met all the required dose volume constraints (Table 2), plan robustness quantification thresholds, and the prescription criteria (see 2.F subsection).

**Table 2:** Dose volume constraints for organs at risk

Structure	Dose limits (Gy[RBE])
Esophagus	D_33%_ <65; D_67%_ <55, D_whole volume_ ≤45, as low as reasonably achievable
Liver	D_whole volume_ ≤25; D_50%_ ≤35
Total normal lung	V_20 Gy[RBE]_ <37% is desirable; V_20 Gy[RBE]_ >41% is a major deviation
Spinal cord	D_max_ ≤50
Heart	D_33%_ ≤60; D_67%_ ≤45; D_whole volume_ ≤30, as low as reasonably achievable; V_50_ <25%; D_mean_ <20
Skin	D_max_ ≤55 (decided by the treating physician)

In VMAT treatment planning, PTV was used for plan optimization. We applied photon optimizer (PO) model in the Eclipse™ for VMAT optimization, and analytical anisotropic algorithm (AAA) model for dose calculation. For target coverage, PTV_high_ V_100%_ was at least 95% of prescription dose, and PTV_high_ D_0.03 cc_ was not more than 110% of prescription dose. Most commonly, two or three arcs were used.

### 2.E Plan quality evaluation

We calculated CTV D_95%_, D_5%_ (the dose level covering at least 95% and 5% of the structure volume with the highest dose respectively), and D_2 cc_ (the minimum dose for the 2 cc of the structure receiving the highest dose) from the CTV dose‐volume‐histograms (DVH). CTV D_95%_, D_5%_‐D_95%_ and D_2 cc_ were used to indicate CTV dose coverage, dose homogeneity, and hot spots, respectively. The CTV was chosen as the target consistent with our clinical practice. The OAR doses evaluated were spinal cord D_max_, esophagus D_mean_, lung D_mean_, and heart D_mean_. In addition, relative volumes such as, total normal lung V_5 Gy[RBE]_ and V_20 Gy[RBE]_, esophagus V_60 Gy[RBE]_, and heart V_50 Gy[RBE]_ were calculated. V_XGy[RBE]_ was defined as the normalized volume receiving a dose of at least X Gy[RBE].

### 2.F Robustness quantification

To evaluate the robustness of IMPT and VMAT plans, we used the DVH band width as a numerical index: the smaller width value means better plan robustness.

For IMPT plans, we considered 12 perturbed scenarios and one nominal scenario. The range uncertainty due to the CT calibration error was assumed to be ±3.5% of the nominal beam ranges, and the isocenter of the patient was rigidly shifted in the antero‐posterior (A‐P), superior‐inferior (S‐I), and right‐left (R‐L) directions by 5 mm, respectively. Combining range and isocenter shift yielded 12 perturbed scenarios.

For VMAT plans, we created six perturbed scenarios and one nominal scenario. The setup uncertainty caused by the rigid shift of the patient isocenter in the A‐P, S‐I, and R‐L directions (±5mm) produced six perturbed scenarios. To generate these uncertainty scenarios, we manually shifted the isocenter (±5mm) and recalculated the VMAT plans in different uncertainty scenarios. DVH curves for these scenarios were determined in our TPS. We exported the DVH curves and calculated the width of DVH band using in‐house developed software. We ensured that in the worst‐case scenario the CTV D_95%_ was at least 95% of the prescription dose in the dose calculations done on all CTs.

### 2.G Interplay effect evaluation

For IMPT treatment, the average energy layer switch time for all 97 energies was 1.91s, ranging from 1.9 to 2.0 s. The average spill length was 7.9s. The average magnet preparation and verification time was 1.93 ms. The effective magnet scanning speed in x‐direction for high/medium and low energy groups were 5.7 and 7.0 m/s, respectively. The effective magnet scanning speed in y‐direction for high, medium, and low energy groups were 17.1, 18.2, and 22.2 m/s, respectively. The proton spill rate in high, medium, and low energy groups were 9.8, 8.1, and 8.5 monitor unit/s (MU/s), respectively.^38^ The field information and delivery durations of IMPT and VMAT plans can be found in the supplementary material (Tables S2 and S3).

**Table 3:** The comparison of plan quality using DVH indices

DVH index	VMAT	IMPT	*P*‐value
CTV D_2 cc_ (normalized)	105%	106%	0.47
CTV D_5%_–D_95%_ (normalized)	4.4%	4.1%	0.29
Total lung V_5 Gy[RBE]_ (%)	59.98%	**29.39%**	**0.0014**
Total lung V_20 Gy[RBE]_ (%)	24.37%	20.41%	0.51
Total lung D_mean_ (Gy[RBE])	13.56	10.65	0.24
Esophagus V_60 Gy[RBE]_ (%)	1.18%	9.63%	0.47
Esophagus D_mean_ (Gy[RBE])	17.02	20.58	0.14
Heart V_50 Gy[RBE]_ (%)	0.83%	0.83%	0.64
Heart D_mean_ (Gy[RBE])	6.97	**1.60**	**0.017**
Spinal cord D_max_ (Gy[RBE])	38.99	**26.50**	**0.0011**

Bold values represent significant difference between IMPT and VMAT DVH indices.

Iso‐layer repainting was used to mitigate the impact of interplay effects.^35^, ^37^, ^39^ If the respiratory motion amplitude was less than 5 mm, the minimum and maximum MU limits in the proton machine were 0.003 and 0.04 MU, respectively. Otherwise, they were 0.003 and 0.01 MU, respectively. Smaller maximum MU limits thereby enforced a higher number of iso‐layer repainting for these patients to mitigate interplay effects. For our iso‐layer repainting technique, a spot would be split into multiple spots if its intensity was larger than the maximum MU limit and the split spots would be appended at the end of the spot list of the same energy layer and delivered through the iso‐layer repainting. A spot, which is planned to deliver MUs smaller than the minimum MU limit, would be rounded up or down depending on whether the amount of MU was larger or smaller than half of the minimum MU limit. For example, with a minimum/maximum MU limit of 0.003/0.04 MU, a spot of 0.081 MU would be split into two spots of 0.04 MU, and the remaining 0.001 MU would be discarded since it was less than half of the minimum MU limit (0.0015 MU); a spot of 0.042 MU would be split into one spot of 0.04 MU and one spot of 0.003 MU, since the remaining 0.002 MU was larger than 0.0015 MU.

For IMPT plans, we developed software to calculate the dose under the influence of interplay effects.^29^, ^30^, ^40^, ^41^ In the software, time‐dependent spot delivery parameters, 4D CTs, and the time spent in each phase during the 4D CT simulations were used^39^, ^40^, ^42^, ^43^, ^44^ to calculate the dose delivered in a patient with interplay effects considered. We randomized the starting phase of each field per fraction to effectively mitigate the impact of the starting phase.^40^ The results of the DVH indices were presented using median values of the corresponding DVH indices with error bars. The error bars indicate maximum and minimum values of the corresponding DVH indices from all patients. No interplay effect evaluation was done for VMAT plans.^45^, ^46^

### 2.H Statistical analysis

In order to allow for a fair comparison, all IMPT and VMAT plans were normalized to have a CTV D_95%_ of 100% of the prescription dose in the nominal scenario. We used the Wilcoxon rank sum test included in MATLAB^®^ 2013 to compare all evaluation metrics. *P‐*values less than 0.05 were considered statistically significant. The points located outside 1.5 times the interquartile range above the upper quartile and below the lower quartile were considered as maximum and minimum outliers, respectively.

## 3 RESULTS

### 3.A Plan quality

**Figure 1:**
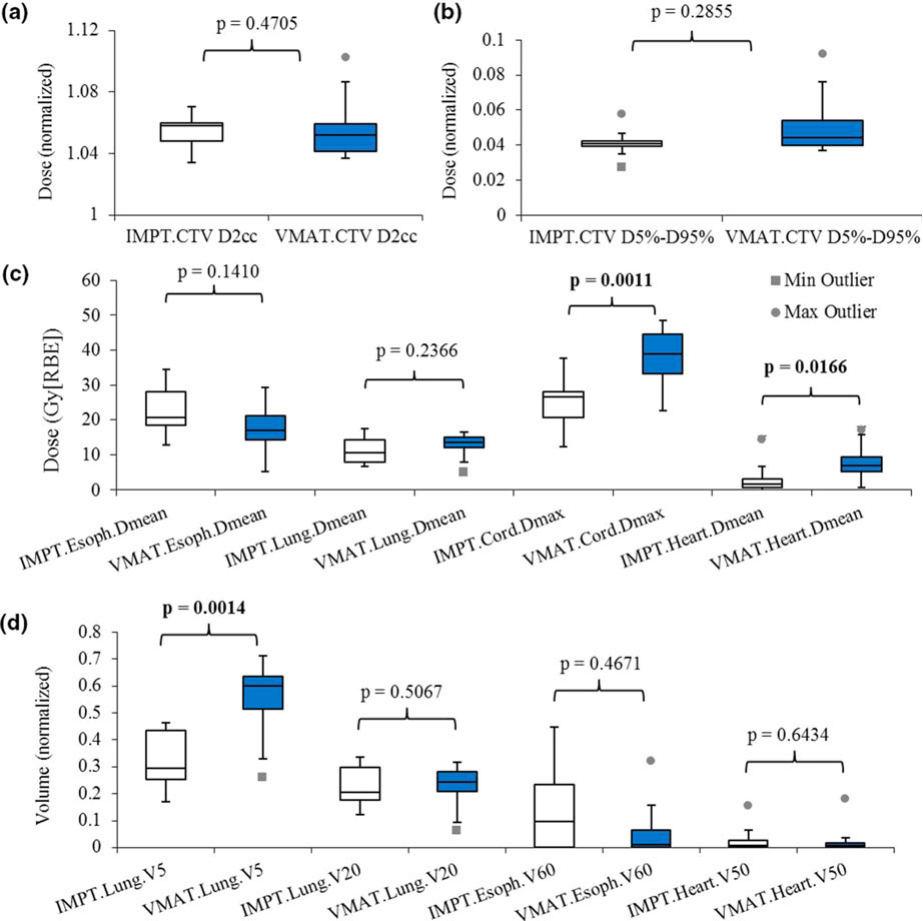
Comparison of the DVH indices between IMPT and VMAT treatment plans. (a) Normalized CTV D_95%_ and D_2 cc_. (b) Normalized CTV D_5%_‐D_95%_. (c) Esophagus D_mean_, lung D_mean_, cord D_max_, and heart D_mean_. (d) Lung V_5 Gy[_ _RBE__]_ and V_20 Gy[_ _RBE__]_, esophagus V_60 Gy[_ _RBE__]_, and heart V_50 Gy[_ _RBE__]_. Numbers at the top of the columns are *P*‐values from Wilcoxon rank sum test. Abbreviations: RBE, relative biological effectiveness.

We compared the plan quality in the nominal scenario (without any uncertainties considered). The IMPT plans performed significantly better in terms of spinal cord D_max_, heart D_mean_, and total lung V_5 Gy[RBE]_ [Figs. 1(a)–1(d), Table 3]. Compared to the VMAT plans, IMPT plans had comparable D_2 cc_ (normalized by the prescription doses), comparable CTV D_5%_‐D_95%_ (normalized by the prescription doses), and comparable protection of most of the other OARs (esophagus D_mean_, total lung D_mean_, total lung V_20 Gy[RBE]_, esophagus V_60 Gy[RBE]_, and heart V_50 Gy[RBE]_) [Figs. 1(a)–1(d), Table 3].

### 3.B Plan robustness

**Figure 2:**
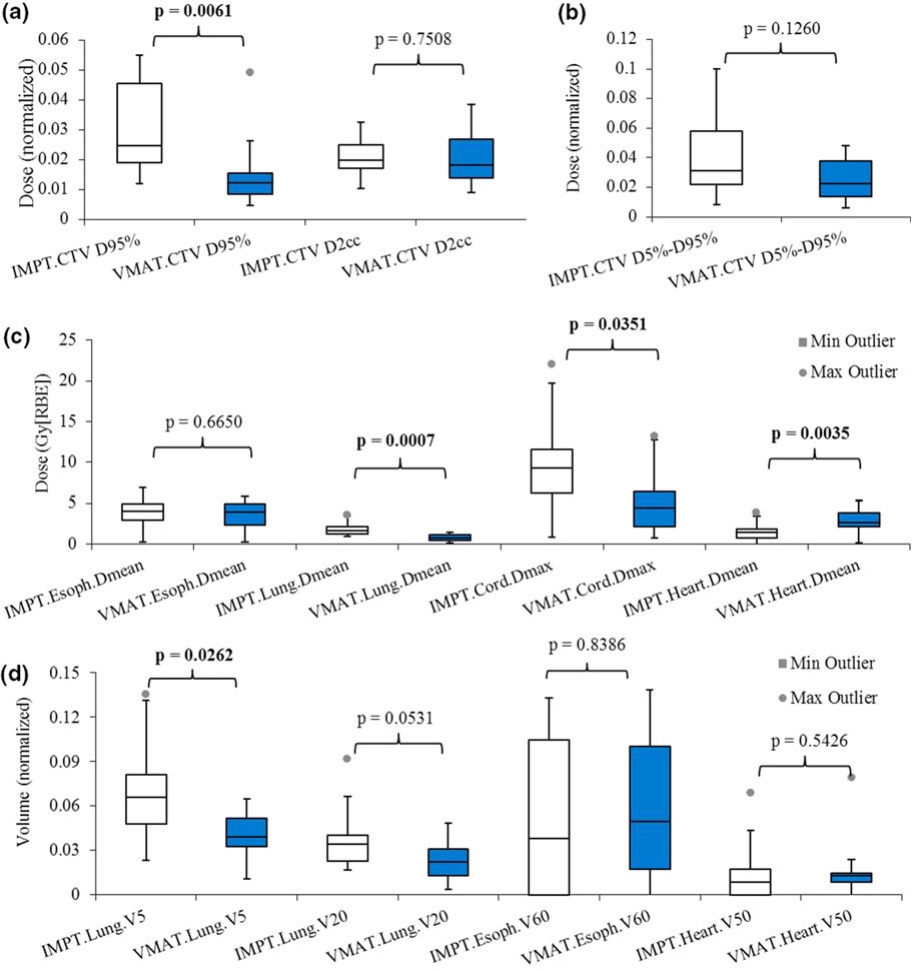
Comparison of plan robustness using the averaged widths from the DVH band method between IMPT and VMAT plans. (a) Normalized CTV D_95%_ and D_2 cc_. (b) Normalized CTV D_5%_–D_95%._ (c) Esophagus D_mean_, lung D_mean_, cord D_max_, and heart D_mean_. (d) Lung V_5 Gy[_ _RBE__]_ and V_20 Gy[_ _RBE__]_, esophagus V_60 Gy[_ _RBE__]_, and heart V_50 Gy[_ _RBE__]_. Numbers at the top of the columns are *P*‐values from Wilcoxon rank sum test. Abbreviations: RBE, relative biological effectiveness.

Figures 2(a)–2(d) displayed the ranges of DVH band widths of CTV and OARs for all 24 patients to indicate plan robustness. *P‐*values are displayed on the top of the columns. The robustness of IMPT plans was statistically better than that of VMAT plans for heart D_mean_, but was statistically worse than that of VMAT plans for CTV D_95%_ (normalized by the prescription doses), spinal cord D_max_, and total lung D_mean_ and V_5 Gy[RBE]_. The robustness of IMPT plans was comparable to that of VMAT plans for D_2 cc_ (normalized by the prescription doses), CTV D_5%_‐D_95%_ (normalized by the prescription doses), esophagus D_mean_, V_60 Gy[RBE]_, total lung V_20 Gy[RBE]_ and heart V_50 Gy[RBE]_ (Table 4).

**Table 4:** The comparison of plan robustness using the width of DVH index bands

DVH index	VMAT	IMPT	*P*‐value
CTV D_95%_ (normalized)	**1.2%**	2.5%	**0.0061**
CTV D_2 cc_ (normalized)	1.8%	2.0%	0.75
CTV D_5%_‐D_95%_ (normalized)	2.3%	3.1%	0.13
Total lung V_5 Gy[RBE]_ (%)	**3.89%**	6.56%	**0.026**
Total lung V_20 Gy[RBE]_ (%)	2.23%	3.41%	0.053
Total lung D_mean_ (Gy[RBE])	**0.79**	1.70	**0.0007**
Esophagus V_60 Gy[RBE]_ (%)	4.96%	3.81%	0.84
Esophagus D_mean_ (Gy[RBE])	3.93	4.00	0.67
Heart V_50 Gy[RBE]_ (%)	1.30%	0.85%	0.54
Heart D_mean_ (Gy[RBE])	2.62	**1.48**	**0.0035**
Spinal cord D_max_ (Gy[RBE])	**4.43**	9.34	**0.035**

Bold values represent significant difference between IMPT and VMAT DVH indices.

### 3.C Interplay effect

**Figure 3:**
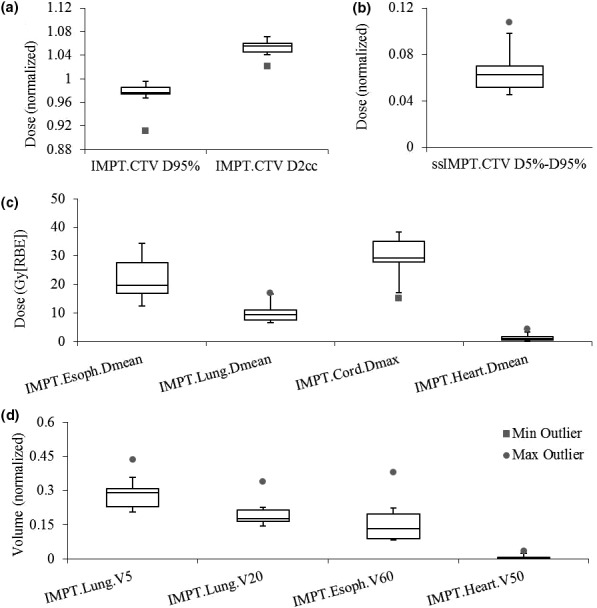
Interplay effect in IMPT evaluated by the dose‐volume histogram indices, including (a) Normalized CTV D_95%_ and D_2 cc_. (b) Normalized CTV D_5%_‐D_95%_. (c) Esophagus D_mean_, total lung D_mean_, spinal cord D_max_, and heart D_mean_. (d) Total lung V_5 Gy[_ _RBE__]_ and V_20 Gy[_ _RBE__]_, esophagus V_60 Gy[_ _RBE__]_, and heart V_50 Gy[_ _RBE__]_. Abbreviations: RBE, relative biological effectiveness.

Interplay effects were only considered for the IMPT plans as shown in Figs. 3(a)–3(d). The median values of CTV D_95%_, D_2 cc_, and D_5%_‐D_95%_ (normalized by the prescription doses) are 0.98, 1.06, and 0.062, respectively. Median values of esophagus D_mean_, total lung D_mean_, spinal cord D_max_, and heart D_mean_ are 19.68 Gy[RBE], 9.38 Gy[RBE], 29.39 Gy[RBE], and 0.94 Gy[RBE], respectively. Median values of esophagus V_60 Gy[RBE]_, total lung V_5 Gy[RBE]_ and V_20 Gy[RBE]_, heart V_50 Gy[RBE]_ are 13.18%, 28.93%, 17.51%, and 0.02%, respectively.

## 4 DISCUSSION

The present study was a treatment planning study, comparing results of small‐spot IMPT treatment planning with results of VMAT treatment planning for patients with stage III NSCLC. Compared to VMAT, IMPT achieved a better protection of spinal cord, heart, and esophagus, and total normal lungs with comparable target homogeneity and hot spot. As for plan robustness, IMPT plans performed better than VMAT in heart D_mean_, and comparable for CTV D_2 cc_, CTV D_5%_‐D_95%_, esophagus D_mean_ and V_60 Gy[RBE]_, lung V_20 Gy[RBE]_ and heart V_50 Gy[RBE]_, but worse for CTV D_95%_, spinal cord D_max_, lung D_mean_ and V_5 Gy[RBE]_.

VMAT gained popularity in the treatment of lung cancer patients due to its high conformality between the prescription iso‐dose lines and targets. IMPT can spare more normal tissues than IMRT, including heart, spinal cord, lung, and esophagus, due to the characteristics of the Bragg peak.^15^ Compared with IMRT, IMPT significantly reduced mean lung dose by 2.8 Gy[RBE] and significantly reduced the lung volumes receiving 5 Gy, 10 Gy, and 20 Gy (p < 0.0001). In our study, IMPT achieved as good plan quality as VMAT in terms of target dose coverage, homogeneity, and sparing of the most OARs. More importantly IMPT significantly lowered heart mean dose, spinal cord maximum dose, and lung V_5 Gy[RBE]_ compared to VMAT. Thus, IMPT may reduce the risks of radiation‐induced cardiac toxicities, neurologic damage, and pneumonitis, and potentially improve the long‐term quality of life of the NSCLC patients.

However, the effectiveness of a treatment plan also depends on plan robustness to both uncertainties and interplay effects. Compared with IMPT, VMAT is more robust with respect to motions or changes in anatomy,^47^ which is consistent with our study. IMPT could be enormously impacted by interplay effects for tumor motions larger than 10 mm and utilization of small‐spot.^28^, ^30^ Interestingly, our results show that IMPT can achieve clinically acceptable plan robustness in the presence of uncertainties. Additionally, with interplay effects considered, the IMPT plans mostly met the clinical requirements except for patient 10. Patient 10 had large amplitude of respiratory motion (11 mm) and a small target (CTV: 47.36 cm^3^). Both would lead to more severe interplay effects.^40^ Due to the proper planning method we used, uncertainties and respiratory motion had limited impact on target coverage and homogeneity, and OAR protection, which is consistent with Inoue et al.^34^ Our results are consistent with Inoue et al.^34^ This is possibly due to the proper planning methods we used. Most of the IMPT plans included in this study were generated using SFO and the rest of them were generated using MFO with robust optimization from a commercial treatment planning system. Li et al.^30^ cautiously extended their IMPT treatment with large spots to lung cancer patients with tumor motion over 5 mm. Our study further extended the applicability of small‐spot IMPT to treat lung cancer patients with tumor motions larger than 5mm but smaller than 11 mm.

The patient groups selected for comparison are not completely statistically comparable. CTV volumes of the patients treated by IMPT are larger than those of the patients treated by VMAT (Table 1). A previous study suggested that large volumes could benefit plan robustness, but increase the difficulties in generating a plan of high quality in the case of IMPT.^48^ In our research, for patients with larger tumor volumes, IMPT still provided better plan quality than VMAT for patients with smaller tumor volumes, and generated treatment plans with clinically acceptable plan robustness. This further supports that new proton centers equipped with proton beam scanning machines with small‐spot may treat locally advanced NSCLC, since IMPT plans are superior to VMAT plans even in a patient group with bigger tumor volumes.

This study has certain limitations. The number of the patients included in this study was small and not matched. A study with a larger patient population with both VMAT and IMPT plans is warranted to generalize our conclusions. Impact from target size and number of repainting remains important topics for future work.

## 5 CONCLUSION

Small‐spot IMPT significantly improves sparing of spinal cord, heart, and lung compared to VMAT and achieves clinically acceptable plan robustness at least for the lung cancer patients included in this study with motion amplitude less than 11 mm. The impact of interplay effects is small if procedures described here are used. This study supports the feasibility of clinical use of small‐spot IMPT to treat certain lung cancer patients.

## CONFLICTS OF INTEREST

The authors declare no conflict of interest.

## Supporting information


**Table S1**. Comparison of the doses to non?target tissues between small?spot IMPT and large?spot IMPT.

**Table S2.** The field, energy, and estimated delivery duration used in VMAT plans.

**Table S3.** The field, energy, and delivery duration in the IMPT plans.

**Data S1.** Comparison of IMPT plan quality with different spot sizes.

**Data S2.** Comparison with reported large spot size IMPT results.
