# Supplementary material for: Small‐spot intensity‐modulated proton therapy and volumetric‐modulated arc therapies for patients with locally advanced non‐small‐cell lung cancer: A dosimetric comparative study
Source: J Appl Clin Med Phys. 2018 Oct 17;19(6):140–8. doi: 10.1002/acm2.12459 (PMC6236833; doi:10.1002/acm2.12459)
Supplement: Supplementary file 1 — Table S1. Comparison of the doses to non?target tissues between small?spot IMPT and large?spot IMPT. [file ACM2-19-140-s001.docx]

Supplemental Table 1. Comparison of the doses to non-target tissues between small spot IMPT and large spot IMPT.

|  |  | Small spot size | Large spot size 1[1] | Large spot size 2[2] |
| --- | --- | --- | --- | --- |
| Total Lung | V_5Gy[RBE]_ (%) | 29.39 (16.93-46.52) | 31.8 (14.4-43.6) | 32.9 (16-48.9) |
|  | V_10Gy[RBE]_ (%) | 25.17 (11.56-41.21) | 28.2 (12.5-40.1) | -- |
|  | V_20Gy[RBE]_ (%) | 20.41 (12.11-33.71) | 23.4 (9.6-33.7) | 23.5 (10.9-35.8) |
|  | V_30Gy[RBE]_ (%) | 14.77 (7.14-28.79) | 18.6 (8.2-26.3) | -- |
|  | Mean (Gy[RBE]) | 10.65 (6.49-17.48) | 13.1 (6.4-18.7) | 15 (5.8-19.3) |
| Con. Lung | V_5Gy[RBE]_ (%) | 3.78 (0-29.31) | 5.7 (0.0-18.8) | -- |
|  | V_10Gy[RBE]_ (%) | 1.80 (0-22.35) | 4.3 (0.0-15.3) | -- |
|  | V_20Gy[RBE]_ (%) | 0.45 (0-13.55) | 2.6 (0.0-10.0) | -- |
|  | V_30Gy[RBE]_ (%) | 0.17 (0-8.02) | 0.8 (0.0-3.6) | -- |
|  | Mean (Gy[RBE]) | 0.71 (0.0032-7.32) | 1.2 (0.0-4.4) | -- |
| Ips. Lung | V_5Gy[RBE]_ (%) | 54.47 (28.84-77.94) | 56.3 (26.8-83.0) | -- |
|  | V_10Gy[RBE]_ (%) | 49.76 (25.87-73.58) | 50.9 (23.2-79.1) | -- |
|  | V_20Gy[RBE]_ (%) | 40.74 (20.60-65.07) | 43.3 (17.8-71.2) | -- |
|  | V_30Gy[RBE]_ (%) | 30.81 (15.99-55.19) | 36.1 (15.2-62.2) | -- |
|  | Mean (Gy[RBE]) | 20.77 (9.58-32.00) | 24.7 (11.8-41.5) | -- |
| Spinal Cord | D_max_ (Gy[RBE]) | 26.17 (12.25-37.62) | 35.9 (17.8-49.0) | 25.9 (0-37.1) |
|  | D_1_ (Gy[RBE]) | 24.08 (7.83-32.48) | 26.8 (4.8-43.0) | -- |
| Heart | V_40Gy[RBE]_ (%) | 2.06 (0-21.95) | 9.3 (0.0-28.0) | -- |
|  | V_30Gy[RBE]_ (%) | 1.10 (0-25.46) | -- | 3.8 (0.2-22) |
| Esophagus | V_40Gy[RBE]_ (%) | 29.8 (0-54.93) | 24.2 (0.0-43.1) | -- |
|  | V_55Gy[RBE]_ (%) | 14.07 (0-50.11) | 16.1 (0.0-30.6) | -- |
|  | V_60Gy[RBE]_ (%) | 14.44 (0-45.74) | -- | 5.8 (0-22.1) |

Abbreviations: RBE=relative biological effectiveness. Con.lung= contralateral lung. Lps.Lung= ipsilateral lung.
