# Supplementary material for: Small‐spot intensity‐modulated proton therapy and volumetric‐modulated arc therapies for patients with locally advanced non‐small‐cell lung cancer: A dosimetric comparative study
Source: J Appl Clin Med Phys. 2018 Oct 17;19(6):140–8. doi: 10.1002/acm2.12459 (PMC6236833; doi:10.1002/acm2.12459)
Supplement: Supplementary file 2 — Table S2. The field, energy, and estimated delivery duration used in VMAT plans. [file ACM2-19-140-s002.docx]

Supplemental Table 2. The field, energy and estimated delivery duration used in VMAT plans.

| Patient number | Field | Energy (MV) | Estimated Delivery Duration  (Minutes) |
| --- | --- | --- | --- |
| 1 | Arcs, 181º CW 20º  Arcs, 20º CCW 181º  Static, 215º  Static, 345º  Static, 90º  Static, 156º | 6  6  6  6  18  18 | 1.4  1.4  0.2  0.1  0.1  0.2 |
| 2 | Arcs, 181º CW 31º  Arcs, 31º CCW 181º  Static, 260º  Static, 168º  Static, 0º | 6  6  6  6  6 | 1.1  1.1  0.1  0.4  0.2 |
| 3 | Arcs, 30º CCW 181º  Arcs, 181º CCW 30º | 6  6 | 1.5  1.5 |
| 4 | Arcs, 179º CCW 0º  Arcs, 0º CW 179º  Static, 158º  Static, 338º | 6  6  6  6 | 1.2  1.2  0.2  0.2 |
| 5 | Arcs, 181º CW 30º  Arcs, 30º CCW 181º  Arcs, 181º CW 30º | 6  6  6 | 1.1  1.1  1.1 |
| 6 | Arcs, 181º CW 30º  Arcs, 30º CCW 181º  Static, 350º  Static, 160º | 6  6  18  18 | 1.5  1.5  0.2  0.2 |
| 7 | Arcs, 181º CW 179º  Arcs, 179º CCW 181º  Static, 205º  Static, 0º | 6  6  18  18 | 2.5  2.5  0.2  0.2 |
| 8 | Arcs, 181º CW 179º  Arcs, 179º CCW 181º  Static, 180º  Static, 0º  Static, 303º | 6  6  6  6  6 | 2.5  2.5  0.1  0.2  0.2 |
| 9 | Arcs, 181º CW 25º  Arcs, 25º CCW 181º  Arcs, 181º CW 25º  Static, 180º  Static, 0º | 6  6  6  18  18 | 2.1  2.1  2.1  0.3  0.4 |
| 10 | Arcs, 179º CW 0º  Arcs, 0º CCW 179º | 6  6 | 1.9  1.9 |
| 11 | Arcs, 181º CW 37.9º  Arcs, 39.2º CCW 181º | 6  6 | 1.5  1.5 |
| 12 | Arcs, 181º CW 179º  Static, 180º  Static, 37º  Static, 297º  Static, 231º  Static, 0º | 6  18  6  6  18  18 | 2.5  0.2  0.1  0.2  0.1  0.1 |

CW: clock-wise; CCW: counter clock-wise
