# Supplementary material for: Small‐spot intensity‐modulated proton therapy and volumetric‐modulated arc therapies for patients with locally advanced non‐small‐cell lung cancer: A dosimetric comparative study
Source: J Appl Clin Med Phys. 2018 Oct 17;19(6):140–8. doi: 10.1002/acm2.12459 (PMC6236833; doi:10.1002/acm2.12459)
Supplement: Supplementary file 3 — Table S3. The field, energy, and delivery duration in the IMPT plans. [file ACM2-19-140-s003.docx]

Supplemental Table 3. The field, energy and delivery duration in the IMPT plans

| Patient number | Field  (gantry, couch) | Energy Range  (MeV) | Estimated Delivery Duration  (Minutes) |
| --- | --- | --- | --- |
| 13 | 70º, 180º  160º, 180º | 73.2~177.5  91.7~183.3 | 3.3  3.0 |
| 14 | 25º, 0º  150º, 0º | 88.5 ~173.6  118.4~200.4 | 2.6  2.2 |
| 15 | 180º, 270º  150º, 180º | 86.9~151.9  93.2~158.3 | 1.8  1.8 |
| 16 | 60º, 180º  145º, 180º  180º, 180º | 71.3~155.1  102.1~169.6  94.8~163.5 | 2.4  2.1  2.2 |
| 17 | 100º, 180º  155º, 180º  180º, 180º | 82.0~167.6  86.9~163.5  80.3~159.9 | 2.8  2.6  2.7 |
| 18 | 150º, 180º  170º, 180º  150º, 0º | 107.8~193.6  96.3~181.4  102.1~193.6 | 2.3  2.4  2.4 |
| 19 | 155º, 0º  170º, 180º | 90.1~151.9  83.7~155.1 | 2.0  2.2 |
| 20 | 180º, 180º  110º, 180º | 100.7~171.6  102.1~183.3 | 2.5  2.5 |
| 21 | 180º, 270º  100º, 0º | 71.3~140.2  80.3~175.6 | 2.1  2.6 |
| 22 | 35º, 180º  165º, 180º  0º, 180º | 91.7~148.1  109.1~175.6  94.8~151.9 | 2.0  2.1  2.1 |
| 23 | 145º, 0º  180º, 180º | 83.7~179.5  76.8~163.5 | 2.9  2.9 |
| 24 | 145º, 0º  160º, 180º | 71.3~189.0  71.3~ 179.5 | 3.8  3.9 |
