# Supplementary material for: Small‐spot intensity‐modulated proton therapy and volumetric‐modulated arc therapies for patients with locally advanced non‐small‐cell lung cancer: A dosimetric comparative study
Source: J Appl Clin Med Phys. 2018 Oct 17;19(6):140–8. doi: 10.1002/acm2.12459 (PMC6236833; doi:10.1002/acm2.12459)
Supplement: Supplementary file 4 — Data S1. Comparison of IMPT plan quality with different spot sizes. [file ACM2-19-140-s004.pdf]

August 29, 2018

Response to the *Journal of Applied Clinical Medical Physics* Submission Liu *et al*:

Ref.: Manuscript #2018-01271A

For "**EDITORIAL TEAM COMMENTS**":

Associate Editor (Remarks to the Author):

I would suggest the author do a quick revision for the following two reasons:

1. There were some disorders of the submitted revision caused by submission system. For example, all the submission contains comments and tracked revision. There is no a clean version to review (but the emailed version really helps). With the minor revision, please make sure the correct version will be submitted.

Reply:
We thank the Associate Editor for pointing this out. We submitted the clean version of the manuscript as "Manuscript Text File – "Article File"", the document of Response to Reviewers 8-29-2018" as "Supplemental Materials – "Response to Reviewers"", and double-checked the file format in submission system.

2. The author also needs to check all references and make sure the consistency on format and information. For example, ref 14, 33, 44, 45, 49 do not have page number; ref 36 and 42 do not have consistent format with others; ref 27 and 40 are not complete. Please carefully go through and correct them.

Reply:
We appreciate your time in reviewing our work in detail. All the references are corrected as suggested.

On behalf of all the authors, again, we sincerely thank you for your time and scientific contribution in the review of this manuscript prospectively. We look forward to hearing from the *Journal of Applied Clinical Medical Physics* Review and Editorial Committee.

Wei Liu, Ph.D.
On behalf of the Radiation Oncology Group investigators
