# Supplementary material for: Small‐spot intensity‐modulated proton therapy and volumetric‐modulated arc therapies for patients with locally advanced non‐small‐cell lung cancer: A dosimetric comparative study
Source: J Appl Clin Med Phys. 2018 Oct 17;19(6):140–8. doi: 10.1002/acm2.12459 (PMC6236833; doi:10.1002/acm2.12459)
Supplement: Supplementary file 5 — Data S2. Comparison with reported large spot size IMPT results. [file ACM2-19-140-s005.docx]

### **Comparison with reported large spot size IMPT results**

We also compared plan quality of IMPT plans with different spot sizes. Compared with the published results of IMPT plans with large spot size from other proton centers^15^, the IMPT with small spots achieved plans with better protection of most OARs, such as lungs, cord, and heart (Supplemental Table 1). In the aforementioned studies, the proton beam scanning machine with large spots had a larger spot sigma (σ) [5-15 mm vs. 2-6 mm, large spot vs. small spot] and spot spacing [6.5-20 mm vs. 5 mm]. As the spot size decreased, the dose distribution penumbrae of the resulting IMPT plans were sharpened, and the dose delivered to normal tissues were reduced. Except esophagus V40Gy[RBE], all the other DVH indices were lower from IMPT plans with small spots (Supplemental Table 1), which agreed with the findings of Moteabbed et al^37^.
